# RELATIONSHIP BETWEEN OCCUPATIONAL FACTORS AND SLEEP DISORDERS AMONG PETROCHEMICAL WORKERS ON HAINAN ISLAND, SOUTH CHINA: A CROSS-SECTIONAL STUDY

**DOI:** 10.13075/ijomeh.1896.02468

**Published:** 2024

**Authors:** Qisheng Wu, Shiheng Fan, Bingxian Zhou, Chunyun Lu, Nengde Zhang, Zhuna Su, Jianye Peng, Dee Yu, Jing Zhang

**Affiliations:** Hainan Medical University, School of Public Health, Heinz Mehlhorn Academician Workstation, Haikou, Hainan, China

**Keywords:** risk factor, sleep disorder, propensity score matching, cross-sectional study, petrochemical enterprise employees, occupational characteristics

## Abstract

**Objectives:**

The study aimed to examine the relationship between occupational factors and sleep disorders among employees in petrochemical enterprises.

**Material and Methods:**

All participants from the employees of Hainan Petroleum Refining and Chemical Enterprises were recruited by the cluster sampling in June 1, 2022. The questionnaire used in this research was primarily composed of 3 sections: demographic characteristics, *Pittsburgh Sleep Quality Index* (PSQI) questionnaire and occupation-related factors affecting sleep disorders. A 1:1 propensity score matching (PSM) method was used to balance the demographic characteristics of the 2 groups. Multivariate logistic regression was employed to analyze the independent factors influencing sleep disorders.

**Results:**

A total of 952 valid questionnaires were collected. The frequency of sleep disorders among the 952 participants was 41.9% (N = 399). After PSM, 356 pairs were successfully matched. There was no statistical difference in socio-demographic characteristics between sleep disorder groups (p > 0.05). The logistic regression analysis showed that employees with weekly working time >40 h exhibited a higher likelihood of sleep disorders (OR: 1.74, 95% CI: 1.09–2.82) than those working ≤40 h. Individuals exposed to high-temperature working environments were more likely to experience sleep disorders (OR: 1.77, 95% CI: 1.12–2.81), while the sleep disorder risk in those with occupational stress was 2.67-fold (OR: 2.67, 95% CI: 1.89–3.80). Employees in storage and transportation (OR: 3.34, 95% CI: 1.81–6.40) and other positions (OR: 1.68, 95% CI: 1.03–2.75) displayed a higher risk of sleep disorders than operational workers.

**Conclusions:**

The frequency of sleep disorders among employees in petrochemical enterprises is high. Weekly working hours, type of work position, high-temperature exposure, and high occupational stress are associated with increased risk of sleep disorders among petrochemical workers. Health policymakers should fully consider these factors in improving the sleep quality of petrochemical workers. Int J Occup Med Environ Health. 2024;37(6):591–601

## INTRODUCTION

Sleep disorders refer to a series of adverse physiological and psychological conditions caused by the inability to fall asleep normally or low sleep quality [1], which is a common risk factor of health conditions [2]. Research has revealed that sleep disorders increase the risk of cardiovascular disease and diabetes while decreasing immune function in the body [3–5]. The frequency of sleep disorders in China, Europe, and America vary due to cultural, lifestyle, healthcare system, and medical resource allocation differences. For example, 30–50% of European adults have experienced varying degrees of sleep disorders [6]. Although the frequency of sleep disorders among Chinese residents was only 19.16% in 2019 [7], this value reached 52.5% among oil-related occupational workers.[8]

Sleep disorders are influenced by various factors, primarily associated with socio-demographic characteristics, lifestyle habits, and environmental factors [9–11]. A study found that the work environments factors has an impact on the frequency of sleep disorders [12]. However, in the study of sleep quality, people tend to largely ignore the work environments factors. Therefore, investigating the correlation between the work environments factors and sleep disorders is crucial.

The petrochemical enterprises in this study are located in the tropics, with climate characteristics such as high temperatures and humidity, and the employees belong to a distinctive professional group. Unlike typical enterprises, petrochemical industry employees are exposed to occupational hazards such as dust, high temperatures, and noise during operational processes [13]. Additionally, the increasing demand for petroleum products in China results in substantial workloads for petrochemical enterprises. The majority of employees are required to work in overtime, leading to prolonged irregular sleep patterns and a heightened susceptibility to sleep disorders [14]. A poor working environment and irregular working hours negatively impact the physical and mental health of workers and increase the risk of disease [15,16]. While some studies have shown that occupational factors affect sleep quality, minimal research is available regarding sleep disorders among petrochemical workers, with an inadequate understanding of the occupational and environmental characteristics influencing sleep disorders in this specific context. Therefore, this study assesses the determinants of sleep disorders among employees in petrochemical enterprises, focusing on the impact of occupational characteristics and environmental factors, aiming to furnish a scientific basis for improving sleep quality.

## MATERIAL AND METHODS

### Participants

A cross-sectional study was conducted in June 2022 at a large-scale petrochemical enterprise in Hainan, China. The inclusion criteria required that the participants:

– were aged ≥ 18 years,
– were employed >1 year,
– were not diagnosed with psychiatric or major respiratory system diseases,
– signed informed consent.

Ultimately, 1129 employees were enrolled for the survey on sleep quality. Of these, 952 (84.32%) available questionnaires were obtained. This study was approved by the Ethics Committee of Hainan Medical University (No. HYLL-2022-247).

### Sample size calculation

The sample size of this study was calculated using a cross-sectional sample size calculation formula:



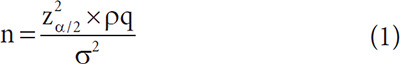


where

ρ − the frequency of sleep disorders,

q − equal to 1–ρ,

δ − the tolerance error, considered as 0.1 р in the present study,

z_α/2_ − the significant test statistic.

When α was set as 0.05, the z_α/2_ was 1.96. According to a previous study, the frequency of sleep disorders among individuals in petroleum-related occupations in China was 52.5% [8]. Considering a potential 20% rate of disqualified questionnaires, this study required a sample size of approx. 500 individuals.

### Questionnaire

This study used a face-to-face survey method involving a paper-based questionnaire to investigate petrochemical industry employees. The questionnaire content included socio-demographic information, *Pittsburgh Sleep Quality Index* (PSQI) questionnaire and occupation-related factors.

The socio-demographic information included sex, age (<30 years, 30–39 years, 40–49 years, and ≥50 years old), nationality (Han, others), marital status (unmarried, married, divorced, or widowed), education (high school and below, special course, undergraduate course, and postgraduate and above), monthly income (<CNY 3000, CNY 3000–4999, CNY 5000–6999, CNY 7000–8999, and ≥CNY 9000), and birthplace (urban, rural). The body mass index (BMI) was calculated using the following formula for Chinese residents. The classifications included:

– underweight (BMI <18.5 kg/m^2^),
– normal (18.5 kg/m^2^ ≤ BMI < 24.0 kg/m^2^),
– overweight (24.0 kg/m^2^ ≤ BMI < 28.0 kg/m^2^),
– obese (BMI ≥28.0 kg/m^2^) [17].

Lifestyle behavior included smoking (no or yes), alcohol use (no or yes), and physical activity (no or yes).

The Chinese version of the PSQI was used to assess sleep quality. There is good reliability and validity in the Chinese version of PSQI [18]. The original scale was designed by Buysse, and was used to measure sleep quality and disturbances over the past month [19]. This scale includes 18 items consisting of 7 components: sleep latency, sleep duration, subjective quality of sleep, use of sleep medication, habitual sleep efficiency, sleep disturbances, and day-time dysfunction. Each item is scored 0–3. The scores of these 7 components were summed to obtain the total PSQI score, with a maximum score of 21. A higher score suggested poorer sleep quality. A reference cut-off score of 7 was used to screen for sleep disorders in Chinese adults.

The occupation-related factors included weekly working hours, working model (regular day shift or shift work), working years (<5 years, 5–15 years, and >15 years), working position (managerial position or ordinary workers), and type of work (operator, storage and transportation workers, and other). The occupational hazards included dust, noise, high-temperature, and organic solvent exposure. Dust exposure is mainly synthetic organic dust, including polyethylene dust, polypropylene dust, etc. Occupational noise exposure was defined as exposure to a noise level ≥80 dB(A) (L_ex,8 h_) or cumulative noise exposure ≥80 dB(A)-years [20]. In this study, the wet bulb globe temperature (WBGT) index instrument was used to measure the physical factors of the workplace according to GBZ/T 189.7-2007 − Part 7: High Temperature in different workplaces of petrochemical enterprises [21]. All monitoring instruments were verified as qualified by the Provincial Bureau of Metrology before use. Wet bulb globe temperature ≥25°C with productive heat source was determined as high temperature exposure [21]. Organic solvent exposure includes ethylene, benzene and benzene series, ethane, etc.

The level of occupational stress was assessed using the effort-reward imbalance (ERI) scale. This scale consists of 23 items, organized into 3 dimensions: effort (6 items), reward (11 items), and overcommitment (6 items). The Likert 5-point scoring method was utilized, with scores ranging 1–5.



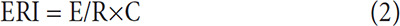



where:

C − the adjustment coefficient, the value was 6/11.

An ERI >1 indicated occupational stress, while an ERI ≤1 suggested its absence. The authors have permission to use this instrument from the copyright holders [22].

### Statistical analysis

The database in this study was established via double-entry using the EpiData 3.0 software. The SPSS 26.0 was employed for data analysis and propensity score matching (PSM) analysis. When the distribution of continuous data deviated from normality, the data were described using the median (Q1; Q3). The differences between the frequency of sleep disorders of the sub-groups were compared using an χ^2^ test.

The PSM was conducted using SPSS v. 26.0 to minimize the differences in the socio-demographic characteristics and lifestyle behavior between the 2 groups and improve the efficiency of the study. A linear regression model was employed with the presence of sleep disorders as the dependent variable and occupational characteristics and hazards as independent variables. Collinearity diagnostics were performed to determine the tolerance and variance inflation factors. The factors influencing sleep disorders were identified via multivariate logistic regression analysis. All analyses were performed using the test level α = 0.05 bilateral.

## RESULTS

### General characteristics of the participants

Table 1 shows the general characteristics of the 952 participants. Across all measurements, the majority was male (85.0%), with 54.5% aged <30 years at the baseline, while the median age was 28 years (IQR: 24–36 years). Most workers (55.0%) reported an undergraduate education, with 47% exhibiting a normal BMI. Non-smokers accounted for 73.3%, while 49.9% did not consume alcohol, and 67.8% engaged in regular physical exercise.

**Table 1.** Demographics, lifestyle behavior of the petrochemical enterprise employees, Hainan Island, South China, 2022

Variable	Participants (N =952)		Me	Q1; Q3
	n	%		
Socioeconomic				
gender				
male	809	85.0		
female	143	15.0		
age			28 years	24 years; 36 years
<30 years	519	54.5		
30–39 years	286	30.1		
40–49 years	105	11.0		
≥50 years	42	4.4		
nationality				
Han	900	94.5		
other	52	5.5		
birthplace				
city	695	73.0		
village	233	24.5		
missing data	24	2.5		
educational level				
high school and below	54	5.7		
special course	321	33.7		
undergraduate course	524	55.0		
postgraduate and above	49	5.1		
missing data	4	0.4		
marital status				
unmarried	532	55.9		
married	404	42.4		
divorced or widowed	15	1.6		
missing data	1	0.1		
monthly income				
<CNY 3000	27	2.8		
CNY 3000–4999	146	15.3		
CNY 5000–6999	273	28.7		
CNY 7000–8999	215	22.6		
≥CNY 9000	274	28.8		
missing data	17	1.8		
Lifestyle				
body mass index				
emaciation	37	3.9		
normal	447	47.0		
overweight	289	30.4		
obesity	172	18.1		
missing data	7	0.7		
smoking				
no	698	73.3		
yes	249	26.2		
missing data	5	0.5		
alcohol use				
no	475	49.9		
yes	452	47.5		
missing data	25	2.6		
physical activity				
no	280	29.4		
yes	645	67.8		
missing data	27	2.8		

### Contains data before and after PSM

Prior to matching, statistically significant differences were observed between the normal sleep and sleep disorders groups in terms of nationality, marital status, and physical exercise (p < 0.05). However, post-matching analyses revealed no significant statistical differences in nationality, marital status, and physical exercise, while remaining gender, age, birthplace, education level, monthly income, BMI, smoking, and Alcohol use continued to show no significant differences (p < 0.05). This highlights the substantial improvement in the balance between both groups after propensity score matching, as outlined in Table 2.

**Table 2.** Comparison of results after propensity score matching (PSM) in petrochemical enterprise employees, Hainan Island, South China, 2022

Variable	Participants [n]							
	before PSM (N = 952)		χ^2^	p	after PSM (N = 712)		χ^2^	p
	normal sleep	sleep disorders			normal sleep	sleep disorders		
Socioeconomic								
gender			0.291	0.590			0.799	0.371
males	467	342			314	306		
female	86	57			42	50		
age			5.899	0.117			0.180	0.981
<30 years	319	200			182	179		
30–39 years	152	134			115	120		
40–49 years	60	45			42	40		
≥50 years	22	20			17	17		
nationality			5.626	0.018			0.223	0.637
Han	531	369			336	333		
other	22	30			20	23		
birthplace			0.826	0.364			0.030	0.862
city	400	295			268	270		
village	142	91			88	86		
educational level			0.544	0.909			1.214	0.750
high school and below	32	22			25	20		
special course	182	139			113	123		
undergraduate course	310	214			201	194		
postgraduate and above	28	21			17	19		
marital status			8.974	0.011			0.623	0.732
unmarried	331	201			185	179		
married	212	192			164	172		
divorce or widowed	9	6			7	5		
monthly income			3.989	0.408			0.306	0.989
<CNY 3000	16	11			8	9		
CNY 3000–4999	93	53			45	48		
CNY 5000–6999	165	108			104	99		
CNY 7000–8999	118	97			87	89		
≥CNY 9000	153	121			112	111		
Lifestyle								
body mass index			2.563	0.464			1.98	0.615
emaciation	19	18			13	18		
normal	261	186			161	163		
overweight	175	114			117	107		
obesity	93	79			65	74		
smoking			1.956	0.162			0.007	0.933
no	414	284			257	256		
yes	135	114			99	100		
alcohol use			0.143	0.706			0.022	0.881
no	278	197			177	175		
yes	259	193			179	181		
physical activity			7.640	0.006			0.025	0.874
no	143	137			120	122		
yes	393	253			236	234		

### The prevalence of sleep disorders

Table 3 presents the univariate analysis results. After adjustment for socio-demographic information and lifestyle behavior via PSM, the weekly working hours, type of work, and occupational stress were statistically different between the sleep-quality groups, while none were evident regarding working mode, working years, working position, dust, noise, high-temperature, and organic solvent exposure. The participants who worked >40 h/week had a higher risk of experiencing sleep disorders than those working ≤40 h/week. The frequency of sleep disorders of storage and transportation workers and other workers were 70.8% and 57.7%, respectively, which exceeded that of operators (46.6%). In addition, the frequency of sleep disorders was higher in participants experiencing occupational stress than those who did not.

**Table 3.** Univariate analysis of sleep disorders in petrochemical enterprise employees, Hainan Island, South China, 2022

Variable	Participants (N = 712) [n (%)]			χ^2^	p
	total	normal sleep (N =356)	sleep disorders (N = 356)		
Working time				8.967	0.003
≤40 h/week	105 (15.3)	67 (19.4)	38 (11.2)		
>40h/week	580 (84.7)	278 (80.6)	302 (88.8)		
Work model				0.021	0.884
regular day shift	328 (47.7)	164 (48.0)	164 (47.4)		
shift work	364 (52.4)	178 (52.0)	182 (52.6)		
Seniority				0.035^a^	0.851
<5 years	340 (47.4)	169 (47.5)	171 (47.2)		
5–15 years	216 (30.1)	110 (30.9)	106 (29.3)		
>15 years	162 (22.6)	77 (21.6)	85 (23.5)		
Position				0.977	0.323
managerial position	125 (17.4)	67 (18.8)	58 (16.0)		
ordinary workers	593 (82.6)	289 (81.2)	304 (84.0)		
Type of work				16.122	<0.001
operator	549 (76.5)	293 (82.3)	256 (70.7)^b^		
storage and transportation worker	65 (9.1)	19 (5.3)	46 (12.7)^b^		
other	104 (14.5)	44 (12.4)	60 (16.6)^c^		
Occupational stress				35.779	<0.001
no	488 (68.5)	279 (79.0)	209 (58.2)		
yes	224 (31.5)	74 (21.0)	150 (41.8)		
Exposure					
dust				1.463	0.226
no	258 (36.6)	136 (38.9)	122 (34.5)		
yes	446 (63.4)	214 (61.1)	232 (65.5)		
noise				0.489	0.484
no	49 (7.0)	22 (6.3)	27 (7.6)		
yes	655 (93.0)	327 (93.4)	327 (92.4)		
high temperature				2.093	0.148
no	126 (17.9)	70 (20.0)	56 (15.8)		
yes	578 (82.1)	280 (80.0)	298 (84.2)		
organic solvent				0.004	0.948
no	212 (30.1)	105 (30.0)	107 (30.2)		
yes	492 (69.9)	245 (70.0)	247 (69.8)		

a Trend χ^2^ value.

b,c The results of pairwise comparison, different letters indicate that the difference between the 2 groups is statistically significant.

### Factors associated with sleep disorders

After PSM adjustment for socio-demographic information and lifestyle behaviors, the statistically significant univariate and harmful environmental factors that might affect sleep disorders during operation were entered into the multivariate logistic regression model to examine the independent constituents influencing sleep disorders. Collinearity diagnosis showed that the tolerance of all the independent variables was >0.1 and the variance inflation factor was <10, indicating no collinearity. The multivariate logistic regression model results indicated that the participants working >40 h/week presented a higher sleep disorder risk than those working ≤40 h/week (OR: 1.740, 95% CI: 1.085–2.816). Furthermore, participants working in high-temperature environments were more likely to experience sleep disorders (OR: 1.765, 95% CI: 1.115–2.807) than those who did not. In addition, the risk of sleep disorders in participants experiencing occupational stress was 2.669-fold that of workers who did not (OR: 2.669, 95% CI: 1.885–3.788). Compared with operators, employees working in storage (OR: 3.337, 95% CI: 1.805–6.350) and transportation and other types of occupations (OR: 1.680, 95% CI: 1.032–2.746) were more likely to experience sleep disorders (Table 4).

**Table 4.** Multivariate logistic regression analysis of sleep disorders in petrochemical enterprise employees, Hainan Island, South China, 2022

Variable	OR (95%CI)	p
Working time		
≤40 h/week	−	−
>40h/week	1.740 (1.085–2.816)	0.022
Exposure		
dust		
no	−	−
yes	1.322 (0.929–1.882)	0.121
noise		
no	−	−
yes	0.533 (0.268–1.062)	0.073
high temperature		
no	−	−
yes	1.765 (1.115–2.807)	0.016
organic solvent		
no	−	−
yes	0.890 (0.620–1.277)	0.527
occupational stress		
no	−	−
yes	2.669 (1.885–3.788)	<0.001
Type of work		
operator	−	−
storage and transportation worker	3.337 (1.805–6.350)	<0.001
other	1.680 (1.032–2.746)	0.037

## DISCUSSION

Analysis showed a sleep disorder incidence of 41.9% in petrochemical workers. The authors study found that the longer the weekly working hours of petrochemical enterprise employees, the higher the frequency of sleep disorders. Workers exposed to high-temperature environments presented a higher risk of experiencing sleep disorders than those who were not. In addition, a strong correlation was evident between occupational stress and sleep disorders. The risk of sleep disorders was higher in employees exposed to long-term occupational stress. Storage and transportation workers and those involved in other types of work had a higher risk of experiencing sleep disorders than operators.

A study [23] on sleep disorders in Mexican oil refining workers revealed a frequency of 34.8%, indicating that the sleep disorders in the petrochemical workers in this study were more serious. Chinese individuals may be more prone to overlooking sleep disorders prioritizing work and social activities, consequently impeding the alleviation of sleep-related issues. Furthermore, the petrochemical enterprises in this study are located in tropical areas, presenting constant high-temperature and -humidity climatic conditions. In addition, the high demand for petroleum products in China has increased the workload of petrochemical workers, resulting in overtime and shift work. Finally, during the petroleum refining process, these workers are exposed to various harmful factors, such as high temperatures, noise, and other toxic substances, severely affecting sleep duration and quality. The authors found that working in a high temperature environment increases the frequency of sleep disorders. This is consistent with the results of a study in South Korea [24]. Working in a high-temperature environment for a long time leads to imbalanced body temperature regulation and stress response, increasing cortisol levels [25]. A rise in basal cortisol levels may lead to sleep disorders [26]. This study examined the association between high temperatures and sleep disorders and its impact on population health. It is suggested that employees wear adequate protective gear during production to reduce the impact of harmful factors and the risk of sleep disorders. The authors’ research on employees in petrochemical enterprises found that long-term occupational stress will increase the frequency of sleep disorders. This is consistent with the results of a study involving office staff in Swedish hospitals [27]. It may be because the petrochemical enterprise employees have special working environment, long working hours and heavy workload, and the corresponding returns do not meet the psychological expectations, resulting in high work pressure. In addition, when employees lack a way of releasing stress, work pressure gradually accumulates and can even cause mental illness. Studies have found that a higher degree of occupational stress increases the concentration of glucocorticoids [28]. Long-term occupational stress leads to excessive hypothalamus-pituitary-adrenal axis activity and higher glucocorticoid levels, affecting the physical and mental health of employees and increasing sleep disorder incidence [29,30]. Employers can convey the harm of occupational stress to employees via publicity, education, and awareness-raising activities. Enterprises should pay attention to the problem of occupational stress among employees, recognize the harm to health, and establish a reasonable performance evaluation mechanism and incentive measures to improve employee morale and reduce its occurrence. The frequency of sleep disorders in storage and transportation workers and other types of work is higher than in operators. Storage and transportation workers in petrochemical enterprises are mainly tasked with storing, loading, and unloading petrochemical products. They also use and maintain organic pump equipment, storage tanks, pipeline valves, and other equipment, as well as judge and deal with production accidents. The loading and unloading times of petrochemical products in petrochemical enterprises vary, leading to irregular sleep patterns and the occurrence of sleep disorders.

This study also presents some limitations. First, the information about sleep disorders was self-reported and prone to reporting bias. Second, the cross-sectional study can only analyze the correlation between sleep disorders and influencing factors, with certain limitations in causal inference. Follow-up studies should conduct cohort studies to comprehensively examine the factors influencing sleep disorders. Finally, data were incomplete for several covariates. The exclusion of workers with missing covariates may result in selection bias and residual confounding by unmeasured covariates. However, the potential for such bias and confounding is low since PSM was used to control the influence of socio-demographic factors and lifestyle on sleep disorders.

## CONCLUSIONS

The results show a high frequency of sleep disorders among employees in petrochemical enterprises, and the risk factors include weekly working hours, high-temperature exposure, occupational stress, and job type. As far as enterprises are concerned, they should pay attention to the sleep disorders of employees and rationalize the workload, such as reasonably arranging the working hours of employees, reducing the physical load, and actively providing pre-job training in mental health.

## References

[1] Han Y, Yuan Y, Zhang L, Fu Y. Sleep disorder status of nurses in general hospitals and its influencing factors. Psychiatr Danub. 2016;28:176–183. 27287793

[2] Amiri S. Sleep quality and sleep-related issues in industrial workers: a global meta-analysis. Int J Occup Saf Ergon. 2023 Mar; 29(1):154–167. 10.1080/10803548.2021.2024376. 34970939

[3] Yin J, Jin X, Shan Z, Li S, Huang H, Li P, Peng X, et al. Relationship of Sleep Duration With All-Cause Mortality and Cardiovascular Events: A Systematic Review and Dose-Response Meta-Analysis of Prospective Cohort Studies. J Am Heart Assoc. 2017;6. 10.1161/JAHA.117.005947. PMC5634263 28889101

[4] Zizi F, Jean-Louis G, Brown CD, Ogedegbe G, Boutin-Foster C, McFarlane SI. Sleep duration and the risk of diabetes mellitus: epidemiologic evidence and pathophysiologic insights. Curr Diab Rep. 2010;10:43–47. 10.1007/s11892-009-0082-x. 20425066 PMC2976532

[5] Besedovsky L, Lange T, Born J. Sleep and immune function. Pflugers Arch. 2012;463:121–137. 10.1007/s00424-011-1044-0. 22071480 PMC3256323

[6] Riemann D, Baglioni C, Bassetti C, Bjorvatn B, Dolenc Groselj L, Ellis JG, et al. European guideline for the diagnosis and treatment of insomnia. J Sleep Res. 2017, 26: 675–700. 10.1111/jsr.12594. 28875581

[7] Wang J, Wu J, Liu J, Meng Y, Li J, Zhou P, et al. Prevalence of sleep disturbances and associated factors among Chinese residents: A web-based empirical survey of 2019. J Glob Health. 2023; 13:04071. 10.7189/jogh.13.04071. 37539543 PMC10401309

[8] Yang F, Zhang Y, Qiu R, Tao N. [Relationship between sleep disorders and hypertension in oil workers in Karamay City]. Wei Sheng Yan Jiu. 2021;50:586–608. 10.19813/j.cnki.weishengyanjiu.2021.04.009. 34311829

[9] Młynarska A, Bronder M, Kolarczyk E, Manulik S, Młynarski R. Determinants of Sleep Disorders and Occupational Burnout among Nurses: A Cross-Sectional Study. Int J Environ Res Public Health. 2022;19 (10):6218. 10.3390/ijerph19106218. 35627754 PMC9140934

[10] Tan X, Saarinen A, Mikkola TM, Tenhunen J, Martinmäki S, Rahikainen A, et al. Effects of exercise and diet interventions on obesity-related sleep disorders in men: study protocol for a randomized controlled trial. Trials. 2013;14:235. 10.1186/1745-6215-14-235. 23886347 PMC3750567

[11] Smith MG, Cordoza M, Basner M. Environmental Noise and Effects on Sleep: An Update to the WHO Systematic Review and Meta-Analysis. Environ Health Perspect. 2022; 130: 76001. 10.1289/EHP10197. 35857401 PMC9272916

[12] Parkes KR. Age and work environment characteristics in relation to sleep: Additive, interactive and curvilinear effects. Appl Ergon. 2016 May;54:41–50. 10.1016/j.apergo.2015.11.009. 26851463

[13] Deng Y, Liu A, Wang D, Li X, Zheng J, Hu W. [Risk assessment of hearing loss of noise workers in three petrochemical enterprises]. Wei Sheng Yan Jiu. 2022;51:918–933. 10.19813/j.cnki.weishengyanjiu. 36539868

[14] Zhou Z, Zhang Y, Sang L, Shen J, Lian Y. Association between sleep disorders and shift work: a prospective cohort study in the Chinese petroleum industry. Int J Occup Saf Ergon. 2022;28: 751–757. 10.1080/10803548.2020.1826706. 33076769

[15] Li X, Gao X, Liu J. Cross-Sectional Survey on the Relationship Between Occupational Stress, Hormone Levels, and the Sleep Quality of Oilfield Workers in Xinjiang, China. Int J Environ Res Public Health. 2019;16. 10.3390/ijerph16183316. PMC6765891 31505823

[16] Ning L, Shi L, Tao N, Li R, Jiang T, Liu J. Effects of Occupational Stress and Circadian CLOCK Gene Polymorphism on Sleep Quality of Oil Workers in Xinjiang, China. Med Sci Monit. 2020;26:e924202. 10.12659/MSM.924202. 32737280 PMC7416614

[17] Chen C. The guidelines for prevention and control of overweight and obesity in Chinese adults. Biomed Environ Sci. 2004;17:1–36. 15807475

[18] Zheng B, Li M, Wang KL, Lv J. [Analysis of the reliability and validity of the Chinese version of Pittsburgh sleep quality index among medical college students]. Beijing Da Xue Xue Bao Yi Xue Ban. 2016;48:424–428. Chinese. 27318902

[19] Buysse DJ, Reynolds CF, 3rd, Monk TH, Berman SR, Kupfer DJ. The Pittsburgh Sleep Quality Index: a new instrument for psychiatric practice and research. Psychiatry Res. 1989; 28: 193–213. 10.1016/0165-1781(89)90047-4. 2748771

[20] Zhou B, Lan Y, Bi Y, Li C, Zhang X, Wu X. Relationship Between Occupational Noise and Hypertension in Modern Enterprise Workers: A Case-Control Study. Int J Public Health. 2022 Nov 4;67:1604997. 10.3389/ijph.2022.1604997. 36405529 PMC9671941

[21] Ministry of Health of the People's Republic of China. Measurement of physical factors in workplace. Part 7: High temperature. GBZ/T189.7-2007 [S]. Beijing: China Standard Press; 2007. Chinese.

[22] Siegrist J, Wege N, Pühlhofer F, Wahrendorf M. A short generic measure of work stress in the era of globalization: effortreward imbalance. Int Arch Occup Environ Health. 2009; 82:1005–1013. 10.1007/s00420-008-0384-3. 19018554

[23] McNamara KA, Robbins WA. Shift Work and Sleep Disturbance in the Oil Industry. Workplace Health Saf. 2023;71:118–129. 10.1177/21650799221139990. 36794861

[24] Park I, Kim S, Kim Y, Yun B, Yoon JH. Association between physical risk factors and sleep disturbance among workers in Korea: The 5th Korean Working Conditions Survey. Sleep Med. 2022;100:157–164. 10.1016/j.sleep.2022.08.011. 36063638

[25] Goodin BR, Smith MT, Quinn NB, King CD, McGuire L. Poor sleep quality and exaggerated salivary cortisol reactivity to the cold pressor task predict greater acute pain severity in a non-clinical sample. Biol Psychol. 2012;91:36–41. 10.1016/j.biopsycho.2012.02.020. 22445783 PMC3606711

[26] Balbo M, Leproult R, Van Cauter E. Impact of sleep and its disturbances on hypothalamo-pituitary-adrenal axis activity. Int J Endocrinol. 2010;2010:759234. 10.1155/2010/759234. 20628523 PMC2902103

[27] Güngördü N, Kurtul S, Erdoğan MS. Evaluation of Sleep Quality, Work Stress and Related Factors in Hospital Office Workers. Med Lav. 2023;114:e2023023. 10.23749/mdl.v114i3.14033. 37309883 PMC10281069

[28] De Sio S, Letizia C, Petramala L, Saracino V, Cedrone F, Sanguigni P, et al. Work-related stress and cortisol levels: is there an association? Results of an observational study. Eur Rev Med Pharmacol Sci. 2018;22:9012–9017. 10.26355/eurrev_201812_16672. 30575947

[29] Gjerstad JK, Lightman SL, Spiga F. Role of glucocorticoid negative feedback in the regulation of HPA axis pulsatility. Stress. 2018;21:403–416. 10.1080/10253890.2018.1470238. 29764284 PMC6220752

[30] Kino T. Stress, glucocorticoid hormones, and hippocampal neural progenitor cells: implications to mood disorders. Front Physiol. 2015;6:230. 10.3389/fphys.2015.00230. 26347657 PMC4541029

